# Readmission risk and costs of firearm injuries in the United States, 2010-2015

**DOI:** 10.1371/journal.pone.0209896

**Published:** 2019-01-24

**Authors:** Sarabeth A. Spitzer, Daniel Vail, Lakshika Tennakoon, Charlotte Rajasingh, David A. Spain, Thomas G. Weiser

**Affiliations:** 1 Stanford University School of Medicine, Stanford, California, United States of America; 2 Stanford Division of General Surgery, Section of Trauma and Critical Care, Stanford, California, United States of America; Monash University, AUSTRALIA

## Abstract

**Background:**

In 2015 there were 36,252 firearm-related deaths and 84,997 nonfatal injuries in the United States. The longitudinal burden of these injuries through readmissions is currently underestimated. We aimed to determine the 6-month readmission risk and hospital costs for patients injured by firearms.

**Methods:**

We used the Nationwide Readmission Database 2010–2015 to assess the frequency of readmissions at 6 months, and hospital costs associated with readmissions for patients with firearm-related injuries. We produced nationally representative estimates of readmission risks and costs.

**Results:**

Of patients discharged following a firearm injury, 15.6% were readmitted within 6 months. The average annual cost of inpatient hospitalizations for firearm injury was over $911 million, 9.5% of which was due to readmissions. Medicare and Medicaid covered 45.2% of total costs for the 5 years, and uninsured patients were responsible for 20.1%.

**Conclusions:**

From 2010–2015, the average total cost of hospitalization for firearm injuries per patient was $32,700, almost 10% of which was due to readmissions within 6 months. Government insurance programs and the uninsured shouldered most of this.

## Introduction

Firearm-related injuries are an important and preventable public health problem in the United States, accounting for over 36,000 deaths and almost 85,000 nonfatal injuries in 2015 [1,2], and over 80% of all firearm deaths in high-income countries between 2003 and 2010 [3]. In fact, the age-adjusted rates of firearm fatalities have recently exceeded the rates of motor vehicle fatalities [1].

Though the number of firearm-related fatalities remained relatively stable over the past decade, the number of nonfatal firearm injuries increased [1]. In addition, it has been suggested that patients with nonfatal firearm injuries likely have ongoing, chronic medical and social problems, classified as “chronic trauma syndrome [4].” These injuries thus likely place a persistent, substantial burden on both patients and the US healthcare system.

Firearm violence is estimated to cost $229 billion annually including both direct costs, such as medical care, and indirect costs, such as lost wages and the impact on quality of life [5]. The lifetime medical cost of firearm injuries are high, estimated to be over $3.3 billion in 2000 [6]. The cost of hospital care alone during the initial admission for firearm-related injuries to the US healthcare system was recently estimated to be $733 million dollars annually [7,8]. However, hospital readmissions are an important contributor to long-term healthcare costs of firearm-related injuries [9].

A recent study estimated that 7% of patients with firearms were readmitted within 30-days of discharge, at a cost of approximately $131 million annually [10]. However, this estimated rate of readmission likely underestimates true readmissions given the semi-chronic nature of such injuries. In addition, this annual readmission cost estimation did not account for Nationwide Readmission Database (NRD) limitations that censor readmissions in December based on the year of initial injury.

We aimed to more accurately determine the risk of readmission of firearm-related injuries and quantify the costs and financial burden of these readmissions in order to estimate costs to the US healthcare system. This information may be used to advise decisions surrounding firearm related public policy.

## Materials and methods

### Data sources

We used the Healthcare Cost and Utilization Project (HCUP) NRD from the Agency for Healthcare Research and Quality for 2010–2015, as updated October 4th, 2017 [11–13] The NRD provides longitudinal information about patients’ initial hospital admission and subsequent readmissions from January 1^st^ through December 31^st^, and provides sample weights that can be used to derive national estimates [14]; it does not provide information about patient readmissions that occur in subsequent calendar years, and cannot be linked to follow patients over multiple years; all patient data within the NRD are de-identified. Our primary outcomes of interest were readmission risk and costs associated with readmission.

### Sample selection

We identified patients with a firearm-related admission using the *International Classification of Diseases*, *9*^*th*^ *Revision*, *Clinical Modification (ICD-9-CM)* codes [15]. Inclusion criteria included diagnosis codes of E922.0–0.3, 0.8, 0.9, E955.0–0.4, E979.4, E985.0–0.4, or E970 [15]. E-codes distinguish emergency conditions, allowing identification of patients admitted within the calendar year for an acute firearm-related injury. All subsequent readmissions were analyzed for identified patients. We did not include patients treated and released from the Emergency Department, as these patients are not included in the NRD. Patients who died upon initial admission were excluded from our readmission analysis.

### Variables and sampling weights

The NRD contains a sample of data from the State Inpatient Database (SID), and provides survey weights that allow conversion of the NRD raw data to nationally representative estimations and revised survey weights that allow the NRD data to be combined [16]. Both weighted and unweighted analyses were performed, including the revised survey weights for readmission data. We identified the inpatient visit associated with an E-code indicative as firearm-related as the initial admission, with each subsequent admission following the initial admission for the next 6 months counted as a readmission. We used the ICD Programs for Injury Categorization Stata module to create standard injury severity scores from diagnosis codes [17].

The NRD weighting variable includes criteria that may change from the initial admission to readmission, such as patient’s age and hospital of admission; as a result, the weighting variable for some patients changed from the weighting obtained at the initial admission. As this accounted for only 3.4% of our total population, and as all patient data contain initial admission variables used for weighting, we used the initial admission variables and demographic information for our analyses.

The NRD contains charges associated with each hospital admission and a corresponding cost-to-charge ratio file for each year to allow for estimations of costs from given charges. Charges, which are highly variable within and between hospitals due to reimbursement issues, can be converted to costs using this given ratio within the NRD. We calculated total costs using this ratio conversion, and inflation adjusted these costs to the 2015 dollar using the Consumer Price Index for Hospital Services [18].

### Handling of readmissions

As the NRD’s readmission data are confined to calendar years, readmissions that occur in years following the initial admission are not captured in a manner that allows linkage. In addition, readmission rates are underestimated within each year depending on the month of initial admission; an individual initially admitted in January has twelve months of potential readmission risk, while an individual admitted in December has, at most, one. The exact degree of underestimation of readmissions and costs that results from calendar-year censoring of the dataset depends on the time course of readmission following an initial presentation for firearm injury, as well as the extent to which readmission costs rise or fall as they become more distant from the initial injury. Additionally, estimates of total readmission rates and costs must consider seasonal variation in firearm injuries.

In order to determine the cause of readmission and whether it was likely related to the original firearm injury, we employed the NRD’s Clinical Classifications Software for ICD-9-CM [19]. This is a software tool developed to collapse ICD-9-CM diagnosis and procedure codes into a smaller number of clinically meaningful categories that can be used in lieu of individual ICD-9-CM codes. All single-level diagnosis CCS categories were evaluated by three independent authors (SS, DV, TGW) and categories that would reasonably be related to a prior firearm injury were identified. Disagreements were resolved by consensus. Readmissions were then analyzed based on CCS codes based on whether they appeared related or unrelated to the original firearm injury. Please see S1 Table for our inclusion criteria.

### Model development and statistical analysis

A model of readmission risk was created based on patients admitted in the first half of the year who had a full 6-month of follow-up, adjusted for the following characteristics from the initial inpatient admission: patient age, gender, patient comorbidities, the index of injury severity, whether the patient underwent an operating room procedure, number of chronic conditions of the patient, disposition status (home vs skilled nursing facility vs rehabilitation facility), length of initial stay, and year.

In the NRD between 2010 and 2015, 93% of all calendar year readmissions following discharges in January-June occurred within 180 days. We limited our analysis estimates of 6-month readmission risk and costs. This allowed for completely unmodeled data analysis for all patients admitted between January and June, five months of unmodeled data and one month of modeled data for patients admitted in July, four months of unmodeled data and one month of modeled data for patients admitted in August and so-forth, sequentially increasing the contribution of modelling for patients discharged in the latter part of the year.

For patients discharged between July and December, we employed a two-step method to estimate the frequency and cost of 6-month readmissions for firearm injuries. First, we estimated the 6-month risk of readmission using a Cox proportional hazards model of readmission risk using readmissions data from patients initially admitted between January and June of each of the study years based on patient demographics and other characteristics of the initial admission for firearm injury. Then, we estimated the expected cost of a readmission visit given those same characteristics using linear regression. We included all readmissions in the dataset in our model, which adjusts for the same characteristics used in the proportional hazards model. Values less than 0.05 were considered significant. Stata SE version 14.1 was used for analyses.

## Results

Between 2010–2015, firearm related injuries accounted for 168,045 initial patient admissions, 155,574 of which survived their initial admission, and over 33,000 hospital readmissions within 6 months of discharge; of those, 54% occurred within 30 days. The demographics and injury characteristics for those who survived at initial admission and for those who were readmitted are shown by payer status at initial admission (Table 1). P-values are shown comparing the differences between payer status groups for each characteristic.

**Table 1:** Demographics and injury characteristics of patients admitted and re-admitted for firearm injuries, 2010–2015.

Payer Status	Medicare	Medicare	Medicaid	Medicaid	Private	Private	Self-Pay	Self-Pay	Other	Other	Total	Total	P Value
	Initial Admission	Readmission	Initial admission	Readmission	Initial admission	Readmission	Initial admission	Readmission	Initial admission	Readmission	Initial admission	Readmission	
Total	9455 (6.1)	1951 (10.0)	48,349 (31.1)	6739 (34.5)	32351 (20.8)	3936 (20.2)	41129 (26.4)	4,092 (21.0)	24291 (15.6)	2803 (14.4)	155574	19521	
Gender	7892 (83.5)												P < 0.0001
Male		9493 (83.7)	41508 (85.9)	5690 (84.4)	27937 (86.4)	3281 (83.4)	38303 (93.1)	3789 (92.6)	22311 (91.8)	2575 (91.9)	137951 (88.7)	16925 (86.7)	
Income Quartile in Zip Code of Residence	4700 (50.6)												P < 0.0001
0-25th percentile	2355 (25.3)	5382 (48.8)	27869 (58.4)	3820 (57.3)	13281 (41.6)	1455 (37.5)	24482 (60.4)	2420 (59.7)	12785 (53.6)	1315 (47.8)	83117 (54.2)	9908 (51.4)	
26th to 50th percentile (median)	1472 (15.8)	2873 (25.9)	10004 (21)	1429 (21.4)	7929 (24.9)	1052 (27.1)	8782 (21.7)	855 (21.1)	5572 (23.4)	726 (26.4)	34643 (22.6)	4551 (23.6)	
51st to 75th percentile	768 (8.3)	1856 (16.3)	6756 (14.2)	971 (14.6)	6650 (20.8)	884 (22.8)	5245 (12.9)	575 (14.2)	3768 (15.8)	512 (18.6)	23892 (15.6)	3287 (17.1)	
76th to 100th percentile		997 (9)	3065 (6.4)	449 (6.7)	4041 (12.7)	492 (12.7)	2037 (5)	205 (5.1)	1716 (7.2)	201 (7.3)	11628 (7.6)	1526 (7.9)	
ISS	6512 (68.9)												P < 0.0001
0-9	1463 (15.5)	6885 (61.2)	27041 (55.9)	3278 (48.6)	20520 (63.4)	2184 (55.5)	25554 (62.1)	2104 (51.4)	14474 (59.6)	1453 (51.9)	94102 (60.5)	10361 (53.1)	
10-15	1213 (12.8)	1614 (14.1)	9943 (20.6)	1144 (17)	6040 (18.7)	689 (17.5)	8924 (21.7)	838 (20.5)	5299 (21.8)	582 (20.8)	31670 (20.4)	3542 (18.1)	
16-25	267 (2.8)	2290 (20.1)	7967 (16.5)	1545 (22.9)	4329 (13.4)	776 (19.7)	5161 (12.5)	862 (21.1)	3504 (14.4)	612 (21.8)	22174 (14.3)	4057 (20.8)	
26 or greater		520 (4.7)	3394 (7)	772 (11.5)	1458 (4.5)	286 (7.3)	1490 (3.6)	288 (7)	1010 (4.2)	155 (5.5)	7618 (4.9)	1558 (8)	
OR Procedure Presence	5862 (62)												P < 0.0001
Yes OR procedure		6485 (57.9)	33367 (69)	5113 (75.9)	22518 (69.6)	3116 (79.2)	26969 (65.6)	3143 (76.8)	16835 (69.3)	2241 (79.9)	105550 (67.8)	14866 (76.2)	
GSW Intent	5336 (56.4)												P < 0.0001
Intent not coded	1680 (17.8)	6352 (56.6)	28341 (58.6)	4015 (59.6)	17874 (55.3)	2297 (58.4)	21838 (53.1)	2225 (54.4)	14862 (61.2)	1749 (62.4)	88252 (56.7)	11395 (58.4)	
Unintentional	817 (8.6)	1759 (15.2)	4273 (8.8)	577 (8.6)	4847 (15)	574 (14.6)	4649 (11.3)	434 (10.6)	2273 (9.4)	248 (8.9)	17722 (11.4)	2163 (11.1)	
Self-Inflicted	1244 (13.2)	1375 (12.2)	852 (1.8)	163 (2.4)	1614 (5)	271 (6.9)	791 (1.9)	125 (3.1)	475 (2)	77 (2.8)	4549 (2.9)	839 (4.3)	
Assault	269 (2.8)	1361 (12)	13336 (27.6)	1800 (26.7)	6982 (21.6)	666 (16.9)	11926 (29)	1061 (25.9)	5956 (24.5)	646 (23)	39444 (25.4)	4399 (22.5)	
Undetermined	108 (1.1)	337 (2.9)	1292 (2.7)	154 (2.3)	732 (2.3)	82 (2.1)	1558 (3.8)	188 (4.6)	452 (1.9)	36 (1.3)	4304 (2.8)	522 (2.7)	
Legal Intervention		125 (1.1)	255 (0.5)	30 (0.4)	300 (0.9)	45 (1.1)	367 (0.9)	58 (1.4)	272 (1.1)	46 (1.7)	1303 (0.8)	202 (1)	
Disposition													P < 0.0001
Died	5367 (56.8)	1857 (16.7)		0 (0)		2 (0.1)		0 (0)		0 (0)		2 (0)	
Routine	2387 (25.2)	5367 (47.1)	37879 (78.3)	4264 (63.3)	25073 (77.5)	2401 (61)	35849 (87.2)	3143 (76.8)	21306 (87.7)	2236 (79.8)	125474 (80.7)	12950 (66.3)	
Transfer to Facility	1570 (16.6)	2387 (21)	4849 (10)	1254 (18.6)	3003 (9.3)	668 (17)	1896 (4.6)	388 (9.5)	1154 (4.8)	226 (8.1)	13290 (8.5)	3156 (16.2)	
Home health	129 (1.4)	1570 (14.1)	4609 (9.5)	1050 (15.6)	4016 (12.4)	835 (21.2)	2274 (5.5)	415 (10.1)	1390 (5.7)	285 (10.2)	13861 (8.9)	2980 (15.3)	
AMA, unknown or missing		129 (1.1)	1011 (2.1)	171 (2.5)	252 (0.8)	30 (0.8)	1093 (2.7)	145 (3.6)	436 (1.8)	55 (2)	2921 (1.9)	433 (2.2)	
Mean Age, years	55.3	56.2	26.4	28.4	31.2	33.7	29.7	30.9	30.8	32.1	30.7	33.3	P < 0.0001
Mean ISS	8.3	8.4	11.1	13.2	9.6	11.5	9.5	12	10.1	11.9	10	11.9	P < 0.0001
Mean Length of stay, days	9.6	13.1	10.6	17.2	8.1	13.3	6.3	10.3	7.5	11.5	8.4	13.8	P < 0.0001
Mean Number of chronic conditions	4.3	4.7	1.9	2.5	1.9	2.4	1.4	1.9	1.5	1.8	1.9	2.5	P < 0.0001

Initial admission is the first admission for all patients with a firearm-related injury, shown number (percent). Readmissions are only those with readmissions within 6 months of discharge; demographics are survey weighted and based on initial admission (p<0.001 for all tests of significance). Those who died upon initial admission are excluded as they were not eligible for our readmission analysis.

For patients who were readmitted, their original payer status (on initial admission) is as follows: Medicare (10.0%), Medicaid (35.9%), private insurance (20.2%), self-pay (20.2%) and other (15.2%), which includes patients not charged and those with alternative forms of insurance.

Table 2 shows the number of patients readmitted once, twice, or three or more times. Of patients who were readmitted once with a condition linked to a firearm injury, 15.6% were readmitted one or more times subsequently.

**Table 2:** Unadjusted number of readmissions.

	Number (Percent)
Only one readmission	17368 (10.4)
Only two readmissions	3609 (2.1)
Three or more readmissions	2094 (1.3)

The number of one, two, and more than two readmissions within 6 months per patient, survey weighted but unadjusted.

Table 3 shows the adjusted 6-month risk of readmission by payer status.

The overall 6-month adjusted readmission risk was 15.6%. Medicare patients had the highest readmission risk at 25.8%, while self-pay patients had the lowest readmission risk at 12.1.

**Table 3:** 6-month readmission risk based on payer status at initial discharge.

Payer Status	Readmission Risk, %	Confidence Intervals
Medicare	25.80	25.23-26.35
Medicaid	17.60	17.39-17.81
Private	15.50	15.23-15.73
Self-pay	12.10	12.03-12.26
Other	13.7	13.53-13.92
Overall	15.6	15.46-15.74

Adjusted and weighted 6-month readmission risk, overall and by payer status.

Costs for all hospitalizations over this 6-year period totaled $5.47 billion, with $517 million (9.5%) due to readmission (Table 4). Government insurance, including Medicaid and Medicare, was responsible for $2.47 billion (45.2%) of all costs over the study period. Medicaid contributed $2.1 billion (38.1%), while Medicare contributed $389 million (7.1%). Self-pay patients accounted for 20.1% of costs, and privately-insured patients accounted for 20.1%. Please see the S2 Table for these results of the Cox proportional hazards model.

**Table 4:** Initial and 6-month readmission costs by payer status for all firearm admissions, 2010–2015.

Payer Status	Initial Inpatient Costs	Readmission Costs	Total Costs
Medicare	$331,803,466.52	$57,298,028.00	$389,101,494.52
Medicaid	$1,881,395,513.92	$201,855,797.00	$2,083,251,310.92
Private	$992,067,749.57	$107,353,022.00	$1,099,420,771.57
Self-pay	$1,014,966,149.49	$83,190,786.00	$1,098,156,935.49
Other	$729,224,553.35	$66,464,446.00	$795,688,999.35
Total	$4,949,457,432.85	$516,162,079.00	$5,465,619,511.85

Initial admission and adjusted readmission costs by payer status for all firearm admissions, survey weighted adjusted to 2015 dollars using the Consumer Price Index for Hospital Services, 2010–2015.

## Discussion

Nearly one in 7 patients discharged from the hospital following a firearm-related injury was readmitted within 6-months; of those readmissions, just over 50% occurred within 30 days. This risk was higher for patients who were older, had higher injury severity scores, had longer length of stay, required an operation, and had a non-routine discharge at their initial admission. In accordance with known epidemiology of firearm violence, Medicare patients were older and were overrepresented in self-harm firearm injuries [20]. Readmissions were noted overwhelmingly to be related to prior firearm injuries based on CCS codes; 98.3% of the first readmission, 98.0% of the second readmission, and 98.7% of the third readmission were noted to be due to related conditions.

The annual average cost of these readmissions was $86 million, which represents 9.5% of total hospitalization costs. Between 2010–2015, hospital costs for firearm-related injuries amounted to $5.5 billion for inpatient admissions. This value underestimates the true hospital cost of firearm related injuries, as it excludes Emergency Department visits not resulting in an admission as well as readmissions that occur after 6 months. It is expensive to treat these patients; the average cost of a patient admitted with a firearm-injury was $32,700. Firearm injuries represent an ongoing, expensive burden to both the patients and the US healthcare system.

Despite similar demographics and initial injury characteristics, self-pay patients had significantly different risks of readmission. Medicaid patients have a readmission risk of 17.6%, while self-pay patients have the lowest readmission risk of 12.1%. It is possible that economic and social factors result in a decreased likelihood of self-pay patients receiving follow up care, such as difficulty covering the high costs. Self-pay patients are often faced with the full charges of their inpatient hospital stays, as there is no third-party negotiator. Furthermore, 84% of these patients reside in zip codes that fall below the 50^th^ income quartile. As the average, unadjusted charge of a self-pay patient’s readmission was $49,087, this likely represents a significant, potentially catastrophic, financial burden and bears further investigation.

This study has multiple limitations, the most important of which are rooted in the way the NRD is organized. The NRD contains only patients who are admitted to the hospital, and thus excludes patients who are treated and released in the Emergency Department or who are treated and expire before admission; these hospital-based costs are not captured. The NRD cannot be linked between multiple years. This leads to not only an underestimation of readmissions between years, but also an underestimation of readmissions within years depending on the month of the initial inpatient admission. Although we attempted to account for this limitation through our use of a survival model, it is likely we are still underestimating readmissions. A 1992 study suggested that approximately 25% of readmissions for firearm-related injuries occurred after 2 years [21]. In addition, NRD charges exclude professional fees, which in prior studies have been shown to average approximately 20% of total acute care payments during an inpatient stay, thus leading to a likely underestimate of total hospital costs [7].

Although E-codes are supposed to be used at the initial inpatient admission for the cause of injury, in practice E-codes are occasionally used inaccurately to identify sequela of the initial admission. This could result in the designation of a hospital readmission as an initial admission, resulting in an overestimation of the number of firearm-related injuries that occur each year and an underestimation of the number of readmissions that occur.

Another study limitation is the inability to link readmissions specifically to the firearm-related injury. We assessed the readmission diagnoses for their likely relationship to the original firearm injury, it is possible that some of these patients were admitted to the hospital for an unrelated incident. Furthermore, we did not determine if these admissions were elective or not. These factors may lead to an overestimation of readmissions due to firearm-related injuries. However, our findings are in line with other recent analyses, and we anticipate that other underestimations would likely surpass this effect, leading to an overall underestimation of costs.

Between 2010 and 2015, the US healthcare system spent over $910 million on the inpatient admissions of firearm related injuries annually, over $85 million of which was spent on readmissions. These costs underestimate the lifetime medical costs of patients with firearm injuries because they do not include emergency services, long term health care, home healthcare, rehabilitation, medications, out of pocket, professional fees, or other non-inpatient medical services. Nor do they include opportunity costs from disability and lost productivity, which are likely even higher than actual medical costs.

The expected total cost of patients admitted following firearm-related injuries is over $35,000 per patient, making them expensive to treat. Over 45% of these costs fall upon government insurance programs, and another 20% on the uninsured. While we did not assess conversion to Medicaid or other government-sponsored insurance programs during readmission, this is likely to occur; a prior study noted that such conversion added an additional 14% cost burden on government insurance for firearm injuries [22].

Overall, this study shows that readmissions are an expensive and underestimated cost to society. This information could be used by policy makers to more effectively guide policy decisions surrounding firearms to reduce the incidence of these devastating injuries. For example, these costs could be used to justify expensive injury prevention programs, or a tax on firearm paraphernalia similar to taxes imposed on cigarettes.

## Supporting information

S1 Table All single level diagnosis CCS categories with inclusion status for readmission analysis as evaluated by three independent authors. (DOCX)

S2 Table Cox proportional hazards model output. (DOCX)
