# Supplementary material for: Readmission risk and costs of firearm injuries in the United States, 2010-2015
Source: PLoS One. 2019 Jan 24;14(1):e0209896. doi: 10.1371/journal.pone.0209896 (PMC6345420; doi:10.1371/journal.pone.0209896)
Supplement: S1 Table — (DOCX) [file pone.0209896.s001.docx]

**Supporting Information Table 1: Readmission Diagnosis Groups Inclusion Criteria**
